# Update on Sjögren’s Syndrome 2018

**DOI:** 10.31138/mjr.29.4.193

**Published:** 2018-12-18

**Authors:** Ourania D. Argyropoulou, Athanasios G. Tzioufas

**Affiliations:** Department of Pathophysiology, School of Medicine, National and Kapodistrian University of Athens, Greece

**Keywords:** Sjögren’s syndrome, pathogenesis, germinal centers, salivary gland ultrasonography, treatment

## Abstract

Primary Sjögren’s syndrome (pSS) is a chronic systemic autoimmune disease with a diverse clinical picture, extending from exocrine involvement to extraglandular manifestations. Although pSS remains a disease of unknown etiology, an interaction between genetic susceptibility and environmental triggers is thought to play a key role in disease initiation and progress. Despite the extensive research during the past years, the pathogenetic mechanisms are still elusive and effective therapeutic intervention is still missing. This review aims to provide an overview of the recent literature on the pathogenesis, clinical features and new therapeutic aspects of pSS.

## INTRODUCTION

Primary Sjögren’s syndrome (pSS) is a disease with a female preponderance, leading potentially to disability and impairment of the quality of life. The disease typically presents with ocular and oral dryness, as a result of mono-nuclear infiltration of lacrimal and salivary glands. Extraglandular manifestations affect approximately 75% of pSS patients and they are classified as non-specific such as arthralgias, arthritis, Raynaud’s phenomenon and fatigue, periepithelial characterized by lymphocytic infiltration around epithelial tissues of parenchymal organs such as kidneys (interstitial nephritis), lungs (small airways disease) and liver (primary biliary cirrhosis), and mediated by deposition of immune complexes due to B cell hyperactivity. The latter include purpura, glomerulonephritis and peripheral nerve involvement. Around 5% of patients that most usually belong in the group of extra epithelial manifestations may develop non-Hodgkin’s lymphoma.

Activation of both innate and adaptive immune responses contributes interactively to disease pathogenesis, with prominent interferon (IFN) signatures identified in both peripheral blood and the affected salivary gland tissues. We performed a Medline search of English language articles published into the PubMed database from 1^st^ January 2018 to 31^st^ October 2018, where 570 articles concerning pSS were found. We will here provide an overview of the literature on the pathogenesis, clinical features and new treatment approaches of pSS.

## NEW INSIGHTS INTO PATHOGENETIC MECHANISMS

Although the etiology of pSS remains poorly understood, it is characterized by exaggerated innate and adaptive immunity. The interaction between genetic susceptibility and environmental triggering factors is thought to activate innate immune system in the early stages of the disease. Genetic studies over the past years identified more than 15 loci for Sjögren’s syndrome (SS) and many of these are shared with other autoimmune diseases, especially Systemic Lupus Erythematosus (SLE). The proinflamma-tory cytokines including interleukin (IL)-1, tumour necrosis factor (TNF) and IL-6 have, for a long time, been found upregulated in salivary gland tissues from pSS patients. IL-1 signalling on brain neurons has been previously postulated as a key event in the pathogenesis of sickness behaviour, a condition resembling chronic fatigue, which occurs frequently in the setting of SS. Another cytokine of the IL-1 family shown to be raised in both serum and salivary gland biopsies of SS patients is IL-33, acting synergistically with IL-12 and IL-23 for the induction of IFNγ secretion by natural killer (NK) and NKT cells.^1^ IL-12/IL35 balance may also represent a part of the pathogenetic cascade in SS.^2^ Serum IL-35 levels have been associated with low disease activity in contrast with serum IL-12p70 levels which were associated with more active disease. In blood cellular subsets, both IL 12p35 and EBV-induced gene protein 3 (EBI3) mRNAs were detected only in B cells, with a trend toward a lower level among patients with pSS.^2^ Nilsson et al. in a study of 20 never-smoking pSS patients showed an increase in BAFF, IL-6 and IL-8 in induced sputum suggesting an ongoing inflammatory disease process in the airways in pSS patients.^3^

It is widely accepted that pSS patients display higher expression of type I and type II IFN-regulated genes in both salivary tissue and peripheral blood. One of the most definitive genome-wide association studies in pSS patients identified single nucleotide polymorphisms in many IFN-inducible genes that are implicated in innate immunity, specifically HLA-alleles, STAT4, IRF5, IL-12A and TNIP1.^4^ Bodewes et al. assessed the relationship between systemic IFN type I and II activity and disease manifestations in pSS and they showed that systemic IFN activation is associated with higher activity only in the ESSDAI biological domain but not in the other domains or the total score. Their data raise the possibility that the ESSDAI biological domain score may be a more sensitive endpoint for trials targeting either IFN pathways.^6^ Moreover, Davies et al. analysed by flow cytometry MAPK/ERK and JAK/STAT signalling networks in peripheral blood mononuclear cells from pSS patients upon stimulation with IFN-2ab. Type I IFN induced gene expression was found to be negatively correlated with INF-2ab induced phosphorylation of STAT3 S727 in T cells and positively with pSTAT1 Y701 in B cells, thus indicating a direct involvement of both pathways in SS pathogenesis.^7^ IFN-inducible protein 16 (IFI16) is an innate immune sensor that forms filamentous oligomers when activated by double-stranded DNA (dsDNA). Antiochos et al. revealed that IFI16 is present in a filamentous state in the target tissue of SS and suggest that this property of DNA-induced filament formation contributes to its status as an autoantigen in SS.^8^

B cell activation is a cardinal feature of pSS manifested as hypergammaglobulinemia, germinal centre formation at the site of salivary gland tissue, RF positivity and raised autoantibody titers against ribonucleoprotein complexes.^1^ Although the pathways inducing B-cell activation in pSS remain partially unknown, many studies have indicated that a variety of genes are involved in B-cell survival, proliferation and signalling. Imegenberg-Kreuz et al. analysed the transcriptome of CD+19 B cells from pSS patients. Among the top upregulated and validated genes were CX3CR1, encoding the fractalkine receptor which is involved in the regulation of B cell malignancies, as well as CCL5/RANTES and CCR1. Increased expression of several members of the TNF superfamily was also identified; TNFSF4/Ox40L, TNFSF10/TRAIL, TNFSF13B/BAFF, TNFRSF17/BCMA as well as S100A8 and –A9/cal-protectin, TLR7, STAT1 and STAT2. Among genes with down regulated expression in pSS B cells were SOCS1 and SOCS3, CD70 and TNFA/P3/A20.^9^ BAFF (also known as TNF ligand superfamily member 13B) is central to the cross-talk between early activation of the innate immune system and the stimulation of auto-reactive B cells. CD27+ memory B cells, marginal zone B cells, plasmablasts and plasma cells are the key subsets of B cells involved in the pathogenesis of pSS.^10^ A recent observational study by Barcelos et al. demonstrated that, in pSS, the presence of lower memory B cells counts was associated with longer disease duration and more active disease. Decreased numbers of memory B-cells clearly discriminated pSS from controls. It remains to be clarified whether Sicca patients with decreased memory B-cells represent pSS and if B-cell profiling could help in the diagnosis of pSS.^11^ An important role also exists for the target tissue (exocrine glands, namely the salivary and lachrymal glands), which promotes local B cell activation; notably via the formation of germinal centre-like structures within the epithelium and plasma cell niches. Continuous stimulation of autoreactive B cells by immune complexes is the first step towards clonal escape and lymphomagenesis associated with pSS.^10^

DNA methylation, histone modification and non-coding RNAs are important contributing factors in the pathogenesis of pSS. Mavragani et al. showed that low risk SS and SLE patients exhibit decreased L1 promoter methylation in minor salivary gland tissues and suggested that lymphoid-specific helicase (LSH), and DNA methyltransferase (DNMT)3A should be investigated as candidate upstream mediators of decreased L1 promoter methylation and increased L1 expression.^12^ Aberrant expression of micro-RNAs (miRNAs) has been linked to essentially all complex autoimmune diseases including pSS.^13^ Wang-Renault et al. performed a large-scale analysis of miRNAs in patients with pSS in sorted peripheral blood-derived T and B cells and demonstrated cell-type specific miRNA expression patterns, potentially related to the pathophysiology of the disease. In CD4 T lymphocytes, -let-7d-3p, -miR-155-5p, miR-22-3p, -miR-30c-5p, -miR-146a-5p, -miR-378a-3p and –miR-28-5p were significantly differentially expressed in both the discovery and the replication cohort.^14^ Johansson et al. suggested in all, that reduced levels of miR-31-5p in T cells of SS patients support auto-immune T-cell responses during chronic type I IFN exposure.^15^ Furthermore, in a cohort of 28 pSS patients, Wang et al. demonstrated reduced expression levels of miR-181 and -16 in labial salivary gland tissues of SS patients. These miRNAs were increased in those who possessed high salivary gland pathological focus score (SGPF), thus suggesting a potential role in SS pathogenesis.^16^ The orphan nuclear receptors retinoic acid-related receptor α and γt (RORα and RORγt) are critical in the development of T helper 17 (Th17) cells, and ROR-specific synthetic ligands have proven efficacy in several mouse models of autoimmunity. Weng et al. showed that the expression of RORα was significantly increased in labial salivary glands (LSGs) of patients with pSS and intensified with the disease stage, as attested by the focus score (FS), showing a similar increasing trend with IL-17A and IL-17RA. SR1001, a selective RORα/γt inverse agonist, significantly improved salivary gland secretory function and relieved sialadenitis in treated mice. These data reveal the potential importance of RORα in controlling the lymphocytic invasion in the salivary glands and suggest that RORα may be a druggable target for treating pSS in the future.^17^ In another study, Shah et al. determined the expression of matrix metalloproteinase 9 (MMP9) and its putative transcription factors ETS1 and LEF1, in labial salivary glands of pSS patients. Their results suggest, for the first time, a concerted increase in ETS1 and LEF1 expression in salivary gland epithelial cells of pSS patients that may contribute as an effector mechanism of salivary gland destruction in pSS.^18^ Peripheral blood programmed cell death protein 1 (PD-1) and its ligand (PD-L1) expressed on the surface of T cells, B cells may also participate in the pathogenesis and development of SS through interactions. Total gluco-sides of paeony (TGP), which may increase the expression of PD-1 and its relevant ligand PD-L1 in the peripheral blood mononuclear cells, may play a role in the pathogenesis and development of SS through the PD-1/PD-L1 pathway, by regulating regulatory T cells/T helper cell 17.^19^

## NEW INSIGHTS INTO DIAGNOSIS, CLINICAL MANIFESTATIONS AND PROGNOSIS

In 2016, the new American College of Rheumatology (ACR)/European League Against Rheumatism (EULAR) classification criteria for pSS were developed to make the classification criteria uniform and improve recruitment for clinical trials, but still do not represent diagnostic criteria and may fail in the recognition of the disease especially in its atypical presentation.^20^ With the exclusion of sialography and salivary gland scintigraphy, the available methods for the evaluation of the oral component of pSS include minor or major salivary gland biopsy (MSGB) and unstimulated salivary flow rate (USFR). The presence of germinal centre-like structures (GCs) in the salivary glands of pSS patients has been generally associated with a more intense clinical disease as reflected by a higher focus score (FS) and increased positivity for anti-SSA/Ro and anti-SSB/La autoantibodies.^5,21^ Their association with the risk of lymphoma development is still under debate. Sène et al. in a cohort of 115 pSS patients showed that the presence of ectopic GC-like structures was associated with a 7.8-fold increased risk of lymphoma occurrence,^22^ when others demonstrate that GC-like structures do not represent a risk factor for non-Hodgkin’s lymphoma (NHL).^21^ The prognostic role of histology for NHL in pSS can be however enriched by further exploratory parameters. Specific attention has been paid to the characterization of the NLRP3 inflammasome axis that seems to identify patients with a more severe disease at a greater risk for lymphomas.^23^ To classify pSS, MSGB is required in patients with a negative anti-SSA/Ro. Although MSGB is the gold standard, new reports are focused on the role of ultrasound as an alternative. Indeed, many studies support the value of salivary gland ultrasonography (SGUS) for assessing major salivary gland involvement in pSS patients. Although SGUS is capable to discriminate the normal from the affected tissue, it is poor for discriminating SS from other diseases mimicking pSS. The main advantage of SGUS is the direct visualisation of structural abnormalities of the salivary glands.^5^ Lee et al. in a cohort of 94 pSS patients showed that the SGUS scoring system is a valuable diagnostic method for pSS. Double seropositivity of anti-Ro/SS-A and La/SS-B along with USFR were independent predictive factors for structural damage of the salivary glands.^24^ Mossel et al. suggested that examination of parotid and submandibular glands on one side was sufficient to predict classification of pSS patients according to ACR/EULAR criteria. To further increase feasibility of SGUS in outpatient clinics worldwide, the authors concluded that only hypoechogenic areas could be scored.^25^ Emerging evidence points out the potential use of SGUS, not only for the diagnosis, but also the follow-up of pSS patients. Gazeau et al. found that none of SGUS abnormalities assessed using a semi-quantitative score changed significantly, during a follow-up of nearly 2 years after an initial evaluation for suspected pSS.^26^ The increased morbidity associated with extraglandular disease points out the need for biomarkers able to reflect disease activity. Positive associations were evident between serum sIL-2R and disease activity, the number of affected organs, and the presence of systemic involvement. sIL-2R also reflects the response to glucocorticoid therapy in the disease.^27^

Although keratoconjunctivitis sicca represents the clinical hallmark of pSS, many other organs and systems may be affected during the course of the disease. Arthralgias and arthritis represent a common finding in pSS patients. A recent meta-analysis showed that the presence of anti-citrullinated protein antibodies (ACPAs) ranges from 3% to 9.9%; however, there is no agreement about their clinical significance. Patients with pSS disclosing ACPAs are prone to develop arthritis as part of the clinical spectrum of the disease, but are also at risk of developing RA.^28^ Regarding pulmonary involvement, small airway disease is the most commonly recognized disorder among symptomatic pSS patients, followed by xerotrachea and interstitial lung disease.^29^ It is well established that pSS patients exhibit higher rates of subclinical atherosclerosis compared to age- and gender-matched individuals. In both SS and SLE patients, traditional risk factors for cardiovascular disease (CVD) do not fully explain the heightened rates of subclinical atherosclerosis seen in these patients. Impaired endothelial function, autoantibodies to endothelial cells, type I Interferon (IFN) activity and neutrophil extracellular traps have been all proposed as potential culprits in autoimmune disease-related atherosclerosis. Antibodies to oxidized Low Density Lipoprotein (ox-LDL) seem to have a crucial role in atherogenesis. Cinoku et al. suggested that antibodies to ox-LDL, possibly resulting from B cell hyperactivity, might exert a protective role in the development of atherosclerosis among patients with pSS.^30^ Moreover, Karageorgas et al. showed, for the first time, a significantly higher risk of subclinical atherosclerosis in patients with pSS with impaired sleep; thus suggesting that clinicians should take psychological disturbances into account when trying to assess and manage the cardiovascular disease risk of pSS patients.^31^ Birnbaum et al. in a cohort of 23 pSS patients showed that males with pSS exhibit increased frequency of small fibre neuropathy which may present as pure or mixed with concurrent large-fibre involvement. In addition, the investigators pointed out the importance of distinguishing between dorsal root ganglia (DRG) versus axonal injury, especially given that mechanisms targeting the DRG may result in irreversible neuronal cell death.^32^ Mucocutaneous lesions may have a prognostic value in pSS patients, since certain findings including purpuric eruptions, urticarial vasculitis, Raynaud’s phenomenon and angular stomatitis have been demonstrated to confer an increased risk for B-cell lymphomas.^5^ The evolution into B-cell lymphoma represents one of the main causes of increased morbidity in pSS and occurs in about 5% of patients. Lymphomas of the salivary glands are predominantly B-cell type, including extranodal marginal zone B-cell lymphoma of the mucosa-associated lymphoid tissue (MALT). Parotid and submandibular salivary glands are the most frequent locations of MALT in pSS. Orbits, naso-pharynx, stomach, thyroid and lung are mucosal sites that could also be affected.^33^ Several clinical (persistent enlargement of salivary glands, lymphadenopathy, cryobulinemic vasculitis, peripheral neuropathy, glomerulonephritis, Raynaud’s phenomenon), serological (leukopenia, low C4, monoclonal gammopathy, cryobulinemia, anti-SSA, anti-SSB, rheumatoid factor) and histopathological (high focus score values) features have been proposed as predictors of lymphoma in pSS patients.^33^ In a recent study, Kapsogeorgou et al. suggested low miR200b-5p levels in minor salivary glands as a novel predictive and possibly pathogenetic mechanism-related factor for the development of SS-associated NHL, since its expression is impaired years before the lymphoma clinical onset.^34^

## NEW INSIGHTS INTO TREATMENT

Over the past years, despite considerable efforts to identify novel drugs, the treatment of pSS has not greatly improved. Standard treatments for sicca symptoms include local symptomatic treatment with tear substitution, oral moisturizers and pilocarpine. Cyclosporine A ophthalmic emulsion 0.05% has been demonstrated to be effective and safe in human clinical trials and reduces signs and symptoms of dry eye disease.^35^ Arthralgias and arthritis are usually treated, as happens in other inflammatory arthritides, with hydroxychloroquine, methotrexate or leflunomide, whereas severe organ manifestations can be successfully treated with high-dose methylprednisolone and cyclophosphamide. Severe cryoglobulinaemic vasculitis may favourably respond to plasmapheresis.^5,36^ The detection of specific auto-antibodies along with hypergammaglobulinaemia and the increased incidence of B-cell lymphoma in pSS patients have suggested a potential role of rituximab, a monoclonal anti-CD20 antibody, by depleting B-cells.^5^ The use of rituximab for sicca symptoms based on the current evidence remains highly debatable, but rituximab as a therapeutic option in systemic involvement is accepted and is recommended in the guidelines of the ACR and the British Society of Rheumatology. Articular, haematological, pulmonary, renal involvement and vasculitis benefited most. Rituximab shows some effect in peripheral neuropathy related to vasculitis and cryoglobulinemia, but not in central neurological manifestations (such as multiple sclerosis-like symptoms) or peripheral non-vasculitis neuropathy.^37^ Fischer et al. in the TRACTISS phase III trial with rituximab in pSS demonstrated minimal but statistically significant improvement in SGUS after rituximab compared with placebo.^38^ Emerging data suggest that hydroxychloroquine, a widely prescribed first-line medication for pSS, has no effect on fatigue and sicca manifestations. However, it is routinely prescribed in pSS female patients during pregnancy; owing to the absence of foetal toxicity, reduction of congenital heart block in SSA positive patients, and the positive effect on pre-term delivery rate.^5^ Exposure to HCQ has also been associated with a reduced risk of cutaneous neonatal lupus (cNL). Among cNL cases, those exposed to HCQ tend to have later onset of rash. Both findings suggest a protective effect of HCQ on cNL.^39^ Although, the abundance of TNF-a in the salivary glands of pSS patients suggested a potential therapeutic target, TNF inhibitors showed low efficacy in pSS implying that TNF-α exert immunoregulatory rather than proinflammatory properties in pSS pathogenesis; a finding that was proved in the laboratory, since TNF-α knockout animals cannot form secondary germinal centres.^40^

New insights into lacrimal and salivary dysfunction indicate novel potential targets for future intervention in pSS. In human and animal models, it has been demonstrated that reduced levels of Cystatin C may contribute to increased Cathepsin S activity. This in turn, may contribute to the degradation of other tear proteins including cystatin C, lactoferrin and salivary IgA.^41^ Inhibition of mTOR, blockage of high mobility group box1 (HMGB)-a pleotropic alarm in contributing to inflammation initiation-in NOD mice and the use of mesenchymal stem cells represent interesting areas of research. Baminercept, a lymphotoxin β receptor IgG fusion protein (LTβR-Ig), has also been used but it failed to significantly improve glandular and extraglandular disease in patients with primary SS, despite evidence from mechanistic studies showing that it blocks LTβR signalling.^42^ The efficacy and safety of Epratuzumab, a CD22-targeted humanized monoclonal IgG antibody, was investigated in patients with systemic lupus erythematosus (SLE) and associated SS. B cell reduction was faster in patients with associated SS. Patients with SLE and associated SS treated with Epratuzumab showed improvement in SLE disease activity, which was associated with reduced B cell numbers and IgM levels.^43^

## CONCLUSION

According to the data found in the literature it can be concluded that pSS is a disabling, slow progressive systemic disorder. Thus, it is essential to identify new pathogenetic mechanisms and early disease predictors. For these reasons, and in consideration of the limited effectiveness of current treatments, considerable efforts are currently being made to identify novel biomarkers and therapeutic targets not only to control clinical manifestations and improve quality of life but also to modify the disease course and the adverse outcomes including lymphoma.
